# On Hemiplegia in Childhood

**Published:** 1915-08-28

**Authors:** 


### ON HEMIPLEGIA IN CHILDHOOD.

At a recent meeting of the Section for the Study of Disease in Children (Royal Society of Medicine) an interesting discussion was conducted in reference to a case of hemiplegia. The patient was a girl of ten years who, when sitting on a chair and apparently in good health, suddenly fell to the ground and was found to be the subject of a complete right hemiplegia. The paralysis involved the face as well as the limbs; it was accompanied by much indistinctness of speech, but there was no loss of consciousness and no convulsions were observed. In the course of a few days the paralysis began to improve, and after three weeks or so had largely disappeared. The question was, What was the nature of the lesion responsible for the hemiplegia? And, to judge from the discussion, a confident answer was a matter of great difficulty.

At the outset it may be noted that there was no doubt that the paralysis was due to an organic lesion and was not a mere functional disturbance. Functional hemiplegia is not altogether unknown in children, but it is decidedly rare. In the present instance it could be excluded with confidence, as in the foot of the paralysed side a definite extensor response was obtained. By some the existence of facial paralysis would be quoted against the diagnosis of functional disease, but even if the rule be allowed there are well-defined exceptions to it. Lastly, it may be noted that there was no anæsthesia; not that anæsthesia always means that an accompanying paralysis is functional, nor that the absence of it necessarily proves organic disease. But the characteristic picture of a functional hemiplegia is the association of the paralysis with hemianæsthesia. Hence the absence of the latter was a further point in the diagnosis of organic disease, though, as already stated, the detection of an extensor response left no room for doubt on this point.

Accepting, then, the diagnosis of a definite lesion, the problem was to identify this. One possible cause of hemiplegia—namely, an intracranial tumour—could be excluded with confidence. The sudden onset of the paralysis and its prompt improvement opposed such an explanation, and the absence of headache, vomiting, and optic neuritis were equally significant. The issue thus appeared to be narrowed down either to some form of vascular lesion or to an attack of encephalitis, and the debate left the question quite unsettled. In regard to the latter view the term "encephalitis" is admittedly hardly capable of exact definition. Largely it has grown out of the experience that now and again a child develops an ocular or a facial paralysis, due presumably to a lesion of the corresponding nuclei at the base of the brain. These nuclei lie in series with the motor nuclei of the grey matter of the spinal cord. An inflammatory or other destructive lesion of the latter is familiar as polio-myelitis, and a similar lesion of the grey matter at the base of the brain came, not unnaturally, to be called polio-encephalitis. Hence the paralyses above mentioned were regarded as examples of infantile paralysis, involving not the spinal centres, but the bulbar or basal centres. But if the grey matter (nerve centres) at the base may be the site of an acute destructive process, may not the same be true of the extensive layer of grey matter spread over the surface of the cerebral cortex? May there not, in other words, be, not only a basal polio-encephalitis, but also a cortical polio-encephalitis? And, assuming that this latter condition involves the motor area of one hemisphere, it is manifest that the result may well be a hemiplegia. But an irritating lesion of the motor cortex will almost certainly mean a lively display of unilateral convulsions precedent to the paralysis. Cases in which this order of events is followed undoubtedly occur; they are commonly called encephalitis, though perhaps it were safer to name them hemiplegia after convulsions. To apply this proposal to the case now under review there was this considerable difficulty—namely, that no convulsions had marked either the onset or the progress of the case. Hence a cortical encephalitis seemed out of the question, while a basal encephalitis could hardly cause a hemiplegia, and in the absence of any paralysis of a cranial nerve there was no ground for assuming its existence. It might, of course, be said that the facial paralysis indicated a basal lesion. But as this paralysis was on the same side as the hemiplegia such a suggestion would not apply. For a right facial nuclear lesion, if it disturbed the pyramidal fibres to the limbs at all, would disturb these prior to their decussation at the medulla, and therefore while causing a right facial paralysis would produce paralysis of the limbs on the left. Hence much could be said against the diagnosis of encephalitis.

In favour of a vascular lesion sudden onset could be quoted. But that a child of ten years should have a cerebral hæmorrhage is hard to believe, and embolism is equally difficult in the absence of all signs of cardiac valvular disease. At the same time it is necessary to say this, that there are substantial reasons for believing that vascular lesions do occur in the cerebral cortex in early life, though their exact nature is hard to determine. The reason for this belief is that now and again there are found in the cortex areas of sclerosis or atrophy, or sometimes a definite cyst, while the patient's record contains a history of convulsive seizures and paralysis in early life. What is the character of the original lesion in these conditions is quite uncertain. In some probably syphilis is an underlying cause, and Sir William Gowers used to teach that thrombosis of the veins was often the initial lesion and was the cause of the hemiplegia. In the case here under discussion one or other of these suggestions might be applied, but always with some degree of doubt, the issue remaining open as between encephalitis on the one hand and a vascular lesion on the other. The practical value of the case is the illustration it affords of the relatively favourable prognosis in hemiplegia occurring in early life—assuming, that is, that a gross lesion such as tumour or abscess can be excluded.

